# Serious adverse events of cell therapy for respiratory diseases: a systematic review and meta-analysis

**DOI:** 10.18632/oncotarget.15426

**Published:** 2017-02-16

**Authors:** Runzhen Zhao, Zhenlei Su, Jing Wu, Hong-Long Ji

**Affiliations:** ^1^ Texas Lung Injury Institute, University of Texas Health Northeast, Tyler, Texas, USA; ^2^ Institute of Lung and Molecular Therapy, Xinxiang Medical University, Xinxiang, Henan, China

**Keywords:** serious adverse events, cell therapy, respiratory diseases, systematic review, meta-analysis

## Abstract

**Background:**

Cell therapy holds the most promising for acute and chronic deleterious respiratory diseases. However, the safety and tolerance for lung disorders are controversy.

**Methods:**

We undertook a systematic review and meta-analyses of all 23 clinical studies of cell therapy. The outcomes were odds ratio (OR), risk difference (RD), Peto OR, relative risk, and mean difference of serious adverse events.

**Results:**

342 systemic infusions and 57 bronchial instillations (204 recipients) of cells were analyzed for acute respiratory distress syndrome (ARDS), bronchopulmonary dysplasia, pulmonary arterial hypertension, silicosis, sarcoidosis, extensively drug-resistant tuberculosis, chronic obstructive pulmonary diseases (COPD), and idiopathic pulmonary fibrosis. The frequency of death in adults from any causes was 71 and 177 per 1,000 for cell therapy and controls, respectively, with an OR of 0.31 (95% CI: 0.03, 3.76) and RD of -0.22 (95% CI: -0.53, 0.09). No significant difference was found for ARDS and COPD. The frequency of deaths and non-fatal serious adverse events of 17 open studies were similar to those of randomized controlled trials. Moreover, serious adverse events of allogenic cells were greater than autologous preparations, as shown by frequency, OR and RD.

**Conclusions:**

We conclude that either infusion or instillation of mesenchymal stem stromal or progenitor cells are well tolerated without serious adverse events causally related to cell treatment. Cell therapy has not been associated with significant changes in spirometry, immune function, cardiovascular activity, and the quality of life.

## INTRODUCTION

Acute inflammatory and chronic fibrotic lung diseases have a high mortality and morbidity worldwide, including acute respiratory distress syndrome (ARDS) [1], bronchopulmonary dysplasia (BPD), pulmonary arterial hypertension (PAH), silicosis, sarcoidosis, extensively drug-resistant tuberculosis (XDRTB), chronic obstructive pulmonary diseases (COPD), and idiopathic pulmonary fibrosis (IPF). In spite of considerable advances in our understanding of pathogenesis and interventional strategy, among these heterogenic diseases, lung infection and COPD account for approximately 11.2% and rank top three leading causes of all deaths globally, in particular in low-income countries. Because of the need for better therapeutics, several preclinical studies have examined the benefit of different stem cell preparations, providing support for the clinical trials of cell therapy [2–7]. However, clinical trials are predominately phase 1 and heterogeneous in the source, preparation, route, dose, duration, variables of outcome, and the nature of diseases. Also, the small number of patients enrolled in each of these clinical trials makes it difficult to evaluate safety and tolerance based on a single trial alone.

Previous systematic review and meta-analyses of the safety and efficacy of cell therapy mainly focused on adult multipotent mesenchymal stem (stromal) cells (MSCs) in animal studies [2–8] or induced hematopoietic stem cell transplantations. MSCs may have therapeutic value for ARDS, COPD, IPF, BPD, and PAH, especially with the scarcity of viable pharmacologic but supportive managements [2–7]. However, significant concerns of the safety of cell-based therapy still exist with limited data from clinical studies.

Whether cell therapy worsens the clinical conditions and causally relates to serious adverse events (SAEs) are inconclusive because there are not enough phase 2 trials to date. Therefore, we carried out a systematic review and meta-analyses to evaluate and summarize the safety of cell therapy in addition to clinical variables critically for these lung disorders.

## RESULTS

### Study characteristics

**Figure 1:**
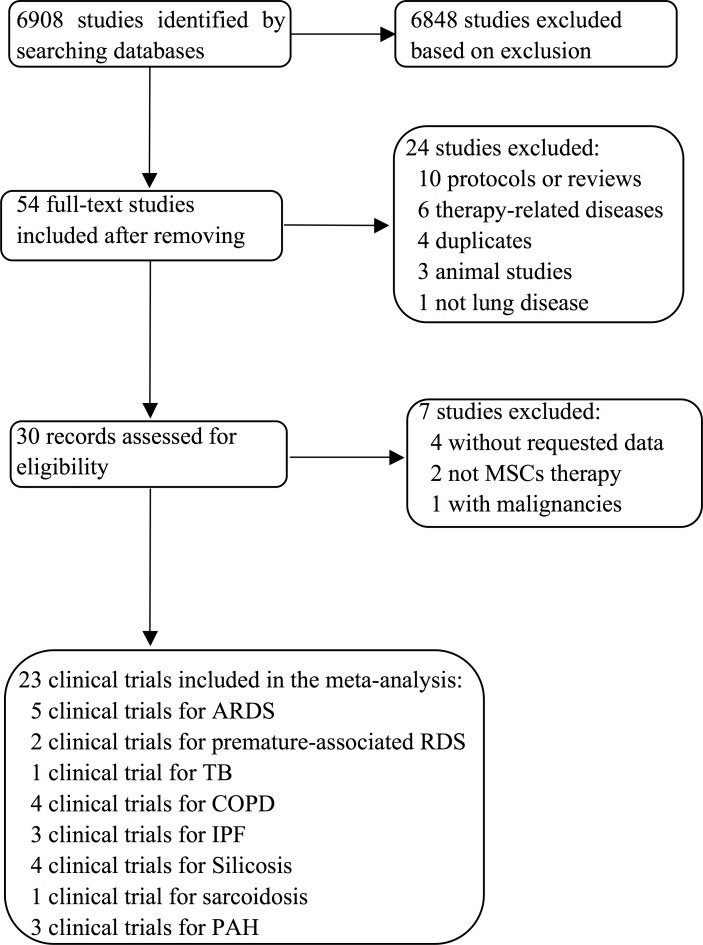
Flow diagram summarizing selection of clinical trials for meta-analysis and systematic review

We collected data from those studies with fully published articles available. The hits from the aforementioned databases of individual reviewer were finally pooled (Figure 1). We analyzed 23 clinical studies of pulmonary diseases: 1) acute lung injury (eight studies, 90 cases), including ARDS (five studies, 37 cases), BPD/RDS of premature infants (two studies, 23 cases), and XDRTB (one study, 30 cases); 2) chronic fibrotic lung injury, including IPF (three studies, 31 cases), silicosis (four studies, 20 cases), COPD (four studies, 77 cases), and sarcoidosis (one study, four cases); and 3) PAH (three studies, 51 cases). Seventeen studies were phase 1a/b, three were phase 2, and three were case series. The profile of included studies is summarized in Table 1. Among them, seven studies were performed in China, three in USA, three in Brazil, two in each of these two countries (Sweden and Korea), and one per country from Russia, Netherland, Canada, Australia, Greece, and Poland. Fifteen studies were registered on the ClinicalTrials.org or other databases. There were four randomized controlled trials (RCT), two nonrandomized trials with controls (nRCT), four dose-escalating trials, six case series, and thirteen open label trials. There were 174 Caucasians, 94 Asians, and five Africans (in total 273 patients, 140 males and 124 females identified). The age ranged from seven day-old pre-term infants to 86 year-old adults, with an average of 43.8 ± 20.7y.

**Table 1:** Main features of included 23 studies

Study	Diagnoses & Severity	Age	Design	Cells (preconditioning, engineering, reprogramming)
Wilson JG et al 2015[20]	9 moderate/severe ARDS	54.9 ± 16.2y	Multicenter, open-label, dose-escalation, phase 1a trial (NCT01775774)	Allogenic BM-MSCs (from NHLBI-PACT), 1, 5, and 10 × 10^6^/kg cultured and re-suspended in Plasmalyte-A (100 ml) for 3 groups, 3 patients/group, 1 dose i.v.
Zheng G et al 2014[10]	12 moderate ARDS (6:6)	66.7 ± 20.4 69.8 ± 9.1y(ctl)	Single center, phase 1/2 (NCT01902082)	Allogenic human AD-MSCs (ATCC, PSC-500-011) expanded in DMEM with 2% FBS, EGF, and FGF. 1 × 10^6^/kg cultured with patients’ serum, 1 dose in saline i.v.
Liu WW et al 2012[11]	13 paraquat-induced ARDS (5:8)	17.8 ± 4.3 22.8 ± 8.7y (ctl)	Single center phase 1/2	Allogenic UBC-SMCs 1 × 10^6^/kg suspended in saline 100 ml, sequentially 5 days for 5 patients, i.v.
Simonson OE et al 2015[33]	2 severe ARDS	49.0 ± 9.0y	Mechanistic uncontrolled case series	Allogenic human BM-MSCs in DMEM with lyzed platelets. 2 × 10^6^/kg in saline + 10% AB plasma via central venous pointed to the right atrium. 1 dose i.v.
Chang Y et al 2014[22]	1 male ARDS	59y, male	Case series	UBC-derived MSCs, 6 passages 1 × 10^6^/kg in saline, 1 dose i.t.
Chang YS et 2014[12]	9 premature RDS	10.4 ± 2.6d	Phase 1 dose-escalation trial (NCT01297205)	Pneumostem, passage 6 allogenic UCB-MSCs (Medipost, Korea), 1 × 10^7^ (2ml)/kg (n=3) mild BPD and doubled dose (4 ml) (n=6, 3 mild, 3 moderate BPD), i.t.
Rudnicki J et al 2015[14]	14 premature RDS (5:9)	Gestation 27.0 ± 0.7w 26.4 ± 2.4w (ctl)	Prospective open-labelled trial (NCT02050971)	14±5ml autologous whole cord blood transfusion vs allogenic RBC (20 ± 10ml) i.v.
Skrahin A et al 2014[31]	30 MDR &XDR TB	30.6 ± 8.8y	Open labeled phase 1 trial (DRKS00000763)	Autologous BMMSCs expanded in IMDM with FBS and 2-mercaptoethanel. 0.2~6 × 10^6^/kg in saline containing 5% human serum albumin, 1 doses i.v.
Weiss DJ et al 2013[9]	62 moderate to severe COPD (30:32)	68.1 ± 7.5 64.1 ± 8.8 (ctl), (40-80y)	Phase 2 trial (NCT00683722)	Allogenic BMMSCs (Prochymals) 5 passages in medium with FBS. 1 × 10^8^ in Plasmalyte-A (150ml) with 5% HSA and 100ml/L DMSO, infused on days 0, 30, 60, and 90 for 30 patients, i.v.
Stolk J et al 2016[17]	7 severe COPD	53.9 ± 6.5y	Prospective open-labelled phase 1 trial (NCT01306513)	Autologous BMMSCs (passage 1-3) in LG-DMEM+10% FCS, 1 or 2 × 10^6^/kg (60-140 × 10^6^ cells) weekly, 2 doses i.v.
Ribeiro-Paes JT et al 2011[15]	4 severe COPD	65.8 ± 7.2 (59-76y)	Unicentric open labelled phase 1 (NCT01110252)	G-CSF 5ug/kg on three consecutive days prior to puncture. Autologous BMMSCs at 1 × 10^8^/mL × 1 dose in 30 ml saline i.v.
Stessuk T et al 2013[16]	4 severe COPD	65.8 ± 7.2 (59-76y).	Unicentric open labelled phase 1 (NCT01110252)	G-CSF 5ug/kg on three consecutive days prior to puncture. Autologous BMMSCs at 1 × 10^8^/mL × 1 dose in 30 ml saline, i.v.
Liu WW et al 2015[27]	4 silicosis (stage I-II)	41.5 ± 6.6 (37-51y)	Non-randomized single-center longitudinal study (NCT01977131)	Autologous BMMSCs in MEM +10% FBS, passage 2 was transfected with HGF (48 h), 2 × 10^6^/kg in 100ml saline, i.v. in 3 consecutive weeks.
Morales MM et al 2015[28]	5 accelerated silicosis	41.0 ± 3.7 (18-50y)	Prospective, non-randomized, single-center longitudinal trial (NCT01239862)	Autologous BMMSCs 2 × 10^7^ in 50ml saline delivered via bronchoscopy into each lobe (i.t), 1 dose.
Liu WW et al 2011[25]	1 silicosis	37y, female	Case series	Autologous BMMSCs, passage 3, transfected with HGF, 5 × 10^7^ cells in 50 ml saline i.v. weekly × 3 wks.
Chen LZ et al 2011[24]	10 silicosis	37.9 ± 9.2 (25-50y)	Prospective, single-center longitudinal study	Autologous BMMSCs in Mesencult medium, passage 3 in 50ml saline, 5 × 10^7^ for 7 patients or 48 h HGF-transfected for 3 patients i.v. weekly × 3 wks.
Chambers DC et al 2014[29]	8 severe IPF	G1 64.1 (62.4-66.5y) G2 66.2 (61.1-71.5y)	Open-label, single center, dose-escalation evaluation phase 1b trial (NCT01385644)	Allogenic placenta derived MSCs propagated in DMEM to passage 2, 4, and 5., 4 patients received 1 (n=4) or 2 × 10^6^/kg (n=4) in PlasmaLyte, i.v.
Tzouvelekis A et al 2013[26]	14 mild to moderate IPF	64.4 ± 7.0y	Prospective, open labelled, phase 1b trial	Autologous ADSCs-SVF were obtained by lipoaspiration, activated by autologous platelet rich protein (PRP) and low level laser irradiation. 0.5 × 10^6^/kg × 3 doses in 5ml saline/lung into lower lobes via endobronchial infusion (i.t.).
Kursova LV et al 2009[19]	9 PF, 2 pneumonitis	32 ± 5y	Prospective, open labelled phase I study	Autologous BMMSCs, 1 or 2 × 10^8^, auto-transplantation, 1 dose i.v.
Baughman RP et al 2015[23]	4 chronic pulmonary sarcoidosis (stage II to III)	47.0 ± 10.8 (40-63y)	Phase 1 trial (NCT01440192)	Placenta-derived allogenic mesenchymal-like cells (PDA-001) 1.5 × 10^7^ × 2 doses in 240ml dextran at a week interval, i.v.
Granton J et al 2015[21]	7 severe PAH	52 ± 20y	Phase 1 dose-escalating trial (PHACeT, NCT00469027)	Early outgrowth EPCs of autologous peripheral blood cultured with combined growth factors and transfected with eNOS. 7, 23, 50 × 10^6^ divided into 3 dose, on continuous days to the right artium. i.v.
Zhu JH et al 2008[18]	13 PAH	11.7 ± 3.5y	Open-labelled pilot trial	Autologous EPCs of peripheral blood were cultured in Medium 199 with autolog-ous serum and VEGF, 0.6 ± 0.33 (0.2~1.3) × 10^7^ × 1 dose in 10ml saline, i.v.
Wang XX et al 2007[13]	31 PAH (15:16)	35 ± 12y 36 ± 9y (ctl)	Phase 2 trial (NCT00257413)	Autologous EPCs from 250 ml peripheral blood cultured in Medium 199 with autologous serum and VEGF, 1.1 ± 0.6 (0.4~2.2 × 10^7^) × 1 dose, suspended in 10 ml saline, i.v.

AD-MSCs, adipose-derived MSCs; BMMSCs, bone marrow-derived MSCs; UBC-MSCs, human umbilical cord-derived MSCs; AADSCs-SVF, autologous adipose derived stromal cells-stromal vascular fraction; CPS, chronic pulmonary sarcoidosis; EPC, autologous endothelial progenitor cells.

### Cell characteristics

Mesenchymal stem (stromal) cells (MSCs) were tested in several studies, including bone marrow derived BM-MSCs in 12 studies (three with allogenic and nine with autologous MSCs), adipose-derived AD-MSCs in one study, placenta derived-MSCs in two studies, and human umbilical cord-derived UBC-MSCs in three studies. Autologous endothelial progenitor cells (EPCs) were used in three studies, and stem cell-containing whole umbilical cord blood was applied in one study. Allogenic MSCs were used for nine trials: six for ARDS, one for COPD, one for IPF, and one for sarcoidosis. Autologous MSC/progenitor cells were for 14 trials: one for BPD, one for TB, three for COPD, four for silicosis, two for IPF, and three for PAH. These stem cells were preconditioned by cultured in medium supplemented with serum and growth factors (*e.g*., EGF, FGF, VEGF, HGF) in ten studies and analyzed platelet in two studies. Two studies used granulocyte-colony stimulating factor (G-CSF) to stimulate autologous BM-MSCs *in vivo*. eNOS transfection and low level laser irradiation were applied in two studies, respectively. Over-expression of HGF by transfection in BM-MSCs was carried out in three studies. The dose for single infusion/instillation was from 1~200 × 10^6^ cells in total or 0.2 ~ 10 × 10^6^/kg body weight per patient. Up to 5 infusions per patient were given. In total, 399 deliveries were performed, either intravenously (342) or intratracheally (57). Two studies followed up to one month or less, ten studies examined patients from two to nine months, and eleven trials visited patients from one to three years. In general, the majority of clinical trials (17 reports) delivered stem cells adjunct with standard or supportive treatments (Table 1).

### Fatal adverse events (death)

**Figure 2:**
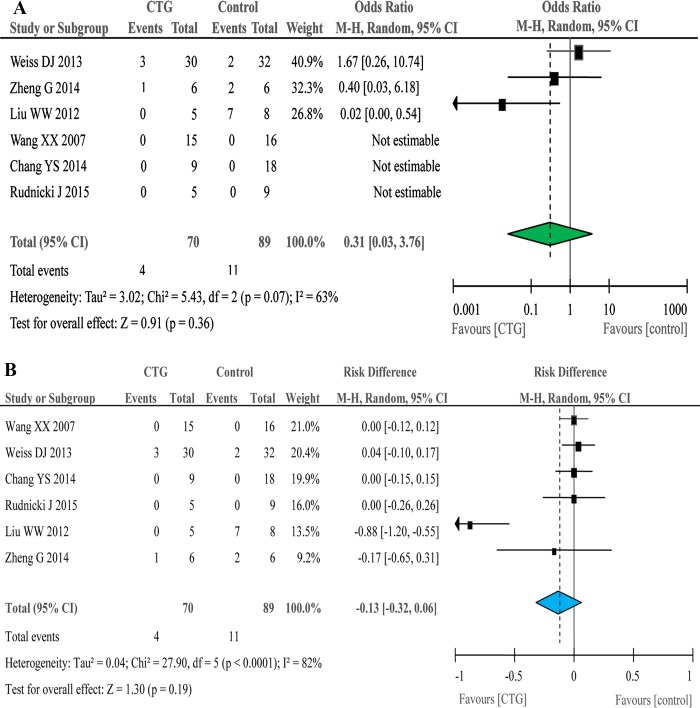
Forest plots for total death in six controlled studies Odd ratio **A**. and risk difference **B**.

All 23 studies considered adverse events (AEs) as primary outcomes. Six studies (three were phase 2 trials) with controls compared all cause-related AEs of cell therapy group with those of controls treated with placebo [9–14]. The frequency of total deaths for any causes of cell recipients was 57 per 1,000, for 4 deaths of 70 patients treated with stem cells were reported (Figure 2A). Eleven deaths of 89 controls were registered with an incidence rate of 124 per 1,000. The odds ratio (OR) of cell therapy and control groups was insignificant, 0.31 (95% CI: 0.03, 3.76) (Table 2). Moreover, we calculated risk difference (RD) between two groups (Figure 2B). A RD value of -0.13 (−0.32, 0.06) for total deaths was obtained, indicating that cell infusions may reduce 32 deaths or increase 6 deaths per 100. We further analyzed the deaths for ARDS and COPD patients separately. The death rate for ARDS was 4% for cell therapy and 22% for controls. However, neither OR (0.1 (95% CI: 0.00, 2.06)) nor RD (−0.25 (95% CI: -0.65, 0.15)) was significant (Table 2). The frequency of deaths for COPD was 100 per 1,000 for treatment group and 62 per 1,000 for controls. Similar to those of ARDS studies, the OR and RD values were insignificant (Table 2). Because there were no deaths in children, the frequency of deaths for adults was thus slightly higher for both cell therapy (71 per 1,000) and controls (177 per 1,000) compared with those of pooled deaths. In contrast, the OR value was identical to that of total deaths of adults and children, and a RD value of -0.22 (95% CI: -0.53, 0.09) was derived (Table 2). For all of deaths were considered as cell therapy-unrelated by the authors, we cannot further analyse cell therapy-caused deaths during the follow-up periods.

**Table 2:** Frequency, odds ratio (OR), and risk difference (RD) of death from any cause for both cell therapy group (CTG) and controls (CTL)

Study	CTG frequency	Control frequency	OR M-H random	p value	RD M-H Random	*p* value
**Total death**	57	124	0.31 (0.03, 3.76)	0.36	−0.13 (−0.32, 0.06)	0.19
**ARDS**	40	220	0.1 (0.00, 2.06)	0.14	−0.25 (−0.65, 0.15)	0.22
**COPD**	100	62	1.67 (0.26, 10.74)	0.59	0.04 (−0.10, 0.17)	0.59
**Adult**	71	177	0.31 (0.03, 3.76)	0.36	−0.22 (−0.53, 0.09)	0.16

Data were analyzed for total, ARDS, COPD, and age.

### Non-fatal SAE

**Figure 3:**
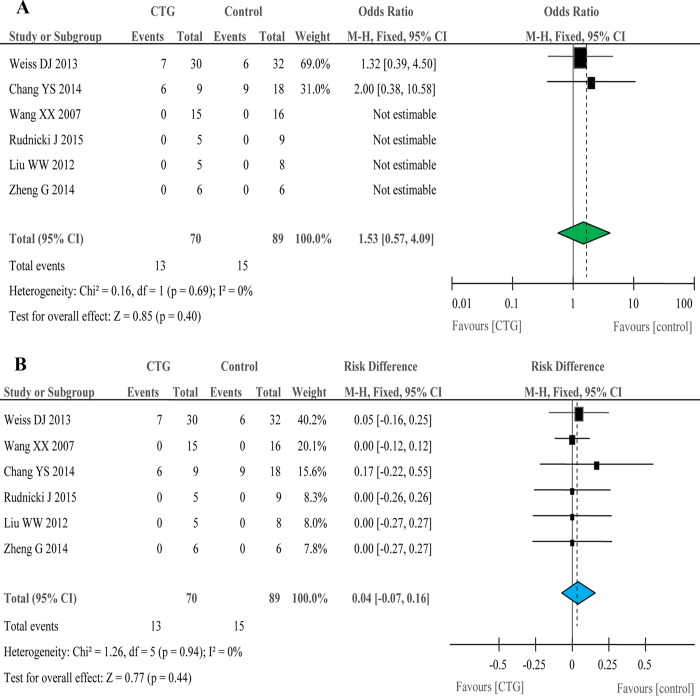
Forest plots for non-fatal serious adverse events in six controlled studies Odd ratio **A**. and risk difference **B**.

The frequency of total non-fatal serious adverse events (SAEs) was 186 per 1,000 for cell therapy and 169 per 1,000 for controls (Table 3). The OR of total non-fatal SAEs was 1.53 (95% CI: 0.57, 4.09), indicating that the probability of non-fatal SAEs for cell therapy was 1.5 fold that of controls (Figure 3A). The RD value was 0.04 (95% CI: -0.07, 0.16), indicating that cell therapy would have 70 less or 160 more non-fatal SAEs per 1,000 (Figure 3B). ARDS recipients had a greater frequency of non-fatal SAEs (240 per 1,000) compared with controls (220 per 1,000) (Table 3). The corresponding OR and RD values were not statistically significant. However, the frequency of non-fatal SAEs for COPD recipients was 1.3 fold (233 per 1,000) that of controls (188 per 1,000). This led to a 45 more non-fatal SAEs per 1,000 in COPD patients receiving cell therapy (Table 3). We then compared non-fatal SAEs between adults and children. The frequency of adults recipients was 125 per 1,000, 1.3 fold that of controls (97 per 1,000). Cell therapy would reduce 100 or add 150 non-fatal SAEs per 1,000 for adult COPD patients (Table 3). Similarly, children recipients also showed a greater frequency of non-fatal SAEs (429 per 1,000) over that of controls (333 per 1,000), resulting in an OR value of 2.00 (95% CI: 0.38, 10.58) and a RD value of 0.11 (95% CI: -0.16, 0.38) (Table 3). Because most of these non-fatal SAEs were therapy-unrelated, and the OR and RD values were insignificant statistically, non-fatal SAEs may not be a concern of cell therapy for respiratory diseases.

**Table 3:** Analyses of total non-fatal SAEs by groups, disease, and age

Study	CTG frequency	Control frequency	OR M-H random	p value	RD M-H random	*p* value
**Total**	186	169	1.53 (0.57, 4.09)	0.40	0.04 (−0.07, 0.16)	0.44
**ARDS**	240	220	2.00 (0.38, 10.58)	0.41	0.07 (−0.12, 0.25)	0.49
**COPD**	233	188	1.32 (0.39, 4.50)	0.66	0.05 (−0.16, 0.25)	0.66
**Adult**	125	97	1.32 (0.39, 4.50)	0.66	0.02 (−0.10, 0.15)	0.70
**Children**	429	333	2.00 (0.38, 10.58)	0.41	0.11 (−0.16, 0.38)	0.43

### Total SAE

The total SAEs combining deaths and non-fatal SAEs showed a less frequency in treatment group (243 per 1,000) than that of controls (292 per 1,000). The OR and RD values were 0.68 (95% CI: 0.15, 3.18) and -0.12 (95% CI: -0.37, 0.13), respectively. For ARDS and COPD patients treated with stem cells, the frequency of total SAEs was 280 per 1,000 and 333 per 1,000, less or greater than controls (for ARDS 439 per 1,000; (for COPD 250 per 1,000). The derived OR value was 0.34 for ARDS and 1.50 for COPD. There were 196 total SAEs per 1,000 for cell recipients and 274 total SAEs per 1,000 for controls in adults. Children showed an identical frequency with non-fatal SAEs for both treatment and control group. Insignificant OR and RD values were computed. Although the OR values for some total SAEs were above 1.0, the causality between all reported total SAEs and cell therapy may not exist.

### Laboratory tests and clinical variables

In addition to AEs aforementioned, cell therapy may affect laboratory tests and clinical variables. We analyzed the mean differences (MD) for spirometry, including FEV1, FVC, and FEV1/FVC (Table 4). The difference between cell therapy and controls was not significant (*p* = 0.42) with a MD value of 0.09 (95% CI: -0.13, 0.31). Similarly, we did not see the significant difference between two groups in immune responses by pooling IL-6, IL-8, SPD, and CRP (*p* = 0.51), in clinical variables by combining LHS, VFD, ICU-free days, SOFA, LIS, 6MWD, Borg dyspnea, DOI, DPAP, and RSS (*p* = 0.97), and in cardiovascular activity and blood tests (*p* = 0.95) (Table 4).

**Table 4:** Summary of laboratory and clinical evaluations

Study	MD (95% CI)	Heterogeneity	Z test
**Lung function tests**	0.09 (−0.13, 0.31)	df = 4 (*p* = 0.93); I² = 0%	Z = 0.81 (*p* = 0.42)
**Clinical evaluation** *	0.01 (−0.48, 0.50)	df = 10 (*p* < 0.0001); I² = 73%	Z = 0.03 (*p* = 0.97)
**Cardiovascular & blood tests**	−0.01 (−0.23, 0.21)	df = 13 (*p* = 0.61); I² = 0%	Z = 0.07 (*p* = 0.95)
**Immune responses**	−0.13 (−0.53, 0.26)	df = 3 (*p* = 0.97); I² = 0%	Z = 0.66 (*p* = 0.51)

Lung function tests include FEV1, FVC, and FEV1/FVC. Clinical evaluations include LHS, VFD, ICU-free days, SOFA, LIS, 6MWD, Borg dyspnea, DOI, DPAP, and RSS. Cardiovascular and blood tests include PaO_2_/FiO_2_, DLCO, SpO_2_, mPAP, PVR, CO, blood pressure, heart rate, hematocrit, glucose concentration, blood pH, Ca^2+^ and K^+^ ions. Immune responses are IL6, IL8, SPD, and CRP. All analyses were performed with the Fixed Effects model except for clinical evaluations (*).

### SAE of phase 1 trials

We also analyzed SAEs in 17 open trials. Five of these 17 studies did not find any AEs [15–19]. Unrelated deaths were reported in four of 17 observational studies [20–23]. Three ARDS patients died of multi-organ failure on day 9, 31, and 118 after cell administrations, which were reviewed and not related to cell treatments^10,12^. One PAH patient collapsed suddenly after discharge who had a history of recurrent presyncope and frequent admission for heart failure^28^. This patient had lowest cardiac output and highest pulmonary resistance. The general reactions to infusions, most often fever, were described in seven studies [23–29].

### Comparison of SAEs between controlled and open-labeled trials

The question raised is whether there were any differences in SAEs between controlled and open-labeled studies. Therefore, we computed frequency, OR, and RD of SAEs between these two types of studies (Tables 5–6). To cross validate these computations, Peto OR and risk ratio (RR) were also calculated for sensitivity analysis. Total SAEs of uncontrolled and controlled studies for ARDS, COPD, and PAH together were 170 and 243 per 1,000 (Table 5). Moreover, the frequency of deaths for uncontrolled studies was 128 vs 57 per 1,000 for controlled studies. Non-fatal SAEs were 43 and 186 per 1,000 for open and RCT trials, respectively. The values of OR, Peto OR, RD, and RR did not show a significant changes for both total SAEs (*p* = 0.35) and deaths (*p* = 0.19). In contrast, the risk of non-fatal SAEs for controlled studies was 4-5 fold that of uncontrolled studies (*p* = 0.04). Furthermore, we compared total SAEs, deaths, and non-fatal SAEs between six controlled and 17 uncontrolled studies for either ARDS, COPD, or PAH separately. The differences between controlled and uncontrolled studies in the frequency of total SAEs (243 vs 333 per 1,000, *p* = 0.78), deaths (40 vs 250 per 1,000, *p* = 0.09), and non-fatal SAEs (240 vs 83 per 1,000, *p* = 0.28) for ARDS were insignificant as reflected by OR, Peto OR, RD, and RR values (Supplement 1). For COPD, one controlled study reported total SAEs (333 per 1,000), deaths (100 per 1,000), and non-fatal SAEs (233 per 1,000)^19^. In sharp contrast, three uncontrolled did not report any SAEs [15–17]. Significant differences were found in total SAEs and non-fatal SAEs but not deaths (Table 6). Further, we did not see marked variance in sex (*p* = 0.66) and race (*p* = 0.78) between controlled and open COPD studies. We also compared SAEs between one controlled [13] and two open-labeled studies [18, 21] for PAH (Supplement 2). Interestingly, none of SAEs was reported in the controlled study [13]. However, the frequency of total SAEs (200 per 1,000), deaths (150 per 1,000), and non-fatal SAEs (50 per 1,000) were described in uncontrolled studies [18, 21]. A significant RD value of -0.20 (95% CI: -0.40, 0.00, *p* = 0.04) was found but not OR, Peto OR, and RR, nor for deaths and non-fatal SAEs.

**Table 5:** Frequency (/1000) of six controlled studies and 17 uncontrolled studies for ARDS, COPD, and PAH

	Controlled (events/total)	Uncontrolled (events/total)	OR (95%CI)	RR (95%CI)	RD (95%CI)	Peto OR	*p* value (OR)
**Total SAEs**	243 (17/70)	170 (8/47)	1.56 (0.61, 3.99)	1.43 (0.67, 3.03)	0.07 (−0.07, 0.22)	1.54 (0.63, 3.77)	0.35
**Death**	57 (4/70)	128 (6/47)	0.41 (0.11, 1.56)	0.45 (0.13, 1.50)	−0.07 (−0.18, 0.04)	0.41 (0.11, 1.52)	0.19
**Non-fatal SAEs**	186 (13/70)	43 (2/47)	5.13 (1.10, 23.92)	4.36 (1.03, 18.46)	0.14 (0.04, 0.25)	3.56 (1.18, 10.71)	0.04

**Table 6:** Frequency, death, and non-fatal SAEs of COPD

	Controlled (events/total)	Uncontrolled (events/total)	OR (95%CI)	RR (95%CI)	RD (95%CI)	Peto OR	*p* value (OR)
Total SAEs	333 (10/30)	0 (0/15)	15.88 (0.86, 292.27)	10.84 (0.68, 173.34)	0.33 (0.15, 0.52)	6.59 (1.51, 28.79)	0.06
Death	100 (3/30)	0 (0/15)	3.95 (0.19, 81.49)	3.61 (0.20, 65.73)	0.10 (−0.04, 0.24)	4.81 (0.41, 56.17)	0.37
Non-fatal SAEs	233 (7/30)	0 (0/15)	9.89 (0.53, 185.97)	7.74 (0.47, 127.11)	0.23 (0.06, 0.41)	5.68 (1.05, 30.81)	0.13

We finally tried to compare the potential contributions of preconditioning/programming and timing. Unfortunately, there were insufficient summarized and individual data to perform these analyses or few data available to obtain meaningful results.

## DISCUSSION

Based on several preclinical studies, cell-based therapies may have value in the treatment for most of common heterogenic lung disorders, including ARDS, COPD, IPF, PAH, silicosis, sarcoidosis, BPD, and pulmonary TRXTB. However, there are insufficient data from phase 2 or 3 trials to analyze the efficacy of cell therapy on each individual respiratory disease. To date, several clinical trials have been completed to address safety. These clinical trials are phase 1 in design with diverse route, source, preconditioning, timing, dose, duration, experimental design, and acute and chronic pulmonary conditions [2–7]. In addition, these studies enrolled a small number of patients, or were even just case reports. It is important to summarize available clinical data to draw valuable clues for the toxicity and potential side-effects of cell therapy. To our knowledge, this analysis of 23 clinical trials represents the first such study to review and analyze pooled data regarding acute and chronic adverse events and potential toxicity for cell-based therapy of the respiratory diseases. This study provides a comprehensive assessment of safety outcomes, including SAEs, variables for the quality of life, spirometry, immune function, cardiopulmonary circulation, and gas exchange for cell-based therapy.

Our results of meta-analysis support the investigators’ conclusions of included clinical trials that SAEs were not causally related to cell-based therapy. The overall OR value for total SAEs from six RCT studies suggests that stem cell therapy is well tolerated with few nonspecific infusion-related reactions. These observations are consistent with the conclusions drawn from the preclinical studies in animal models [2, 4–7]. Of note, the frequency of death in two ARDS studies was numerically less than control, indicating cell therapy may not at least accelerate deterioration of lung diseases. The diversities in age, race, sex, source, preconditioning/programming, route, dose, and disease conditions between included 23 studies suggest cell therapy is well-tolerant and feasible. The safety and tolerance of cell therapy were further supported by our analyses of clinical variables. All of parameters for the quality of life, spirometry, cardiopulmonary function, immune system, and gas exchange are not worsen dramatically.

Well-designed randomized controlled trials maximally limit potential variances associated with the procedures for stem cell preparation and therapy, allocation of patients, and conditions of patients. We thus compared open label and RCT trials. There are no significant differences in total SAEs, mortality, and non-fatal SAEs as shown by OR, Peto OR, RD, and RR analyses between controlled and uncontrolled studies for patients receiving cell therapy, except for non-fatal SAEs. Analyses by diseases identifies COPD patients in one RCT study [9] showed a significant more total SAEs, most likely caused by more non-fatal SAEs compared to those of three uncontrolled studies [15–17]. These studies included patients with similar age and gender, used the same route (i.v.), and followed up from 1-3 years. However, the RCT study applied four doses of allogenic MSCs, while one or two doses of autologous MSCs were infused in three uncontrolled studies. Additional data are required to test whether diverse dose or source causes more non-fatal SAEs in COPD is unknown.

Potential death in animal models of ALI treated with MSCs was systematically reviewed [3]. A significant reduction in the overall odds of death was reported compared to diseased controls without substantial heterogeneity. This decrease in the OD of death was not dependent on pre-specified death time points, gender and species, ALI experimental model, MSC origin and source, route of administration, and MSC preparation. These results are consistent, at least partially with our conclusions drawn from included clinical trials. Unfortunately, other SAEs were not described in the preclinical studies.

Our analysis of overall cell-treated patients from both RCT and open studies suggest that less SAEs, deaths, and non-fatal SAEs are found in patients treated with autologous cells. Because most of reported SAEs were not considered as caused by cell therapy, the reasons for this difference need additional well-designed RCT trials to investigate. If route and dose alter the incidence and mortality of SAEs is still an open question.

Our analysis has several strengths. We had access to data for more than 273 patients receiving cell infusions systematically or instillations intratracheally. We analyzed case series to avoid limitations of literature-based review. We studied clinically relevant subpopulations (e.g., diagnosis, study design, variable, and duration of follow up). We ensured generalization by including studies composed of Americans, Asians, Europeans, Australians, and Africans. To avoid the derivations associated with source, route, culture, gene expression modulation, and dose, we pooled data and analyzed overall alterations. In addition, we crossly validated our analysis with multiple computing models, statistical software, and both meta-analysis and meta-regression.

There are limitations in this study. Studies included in this meta-analysis differed in their methods of recruitment and data collection, and in the preparations of cells, which may explain the heterogeneity between included trials. Nevertheless, we obtained similar results between Peto OR and M-H OR, RR and RD, suggesting the association of cell therapy and safety outcomes is valid. The lack of data regarding the timing and the large range in the duration of follow up might introduce some biases, although the effect is likely to be small. 17 of 23 trials are open labeled without controls, which increases the potential for bias in administration of the intervention and ascertainment of participant-reported outcomes (e.g., clinical scores). Bias may also come from pooling different diseases to compare SAEs associated with route, source, age, gender, and dose. However, by comparable analyses of open-labeled trials with RCT studies or performing sensitivity analyses, we did not identify significant changes in the results. We cannot exclude the differences in SAEs associated with race, age, and gender, but there are insufficient data to compare the contributions of these variables. In addition, there may be fake and false information on the SAEs due to the conflict of interest between the sponsor of clinical trials and patients. More regulatory mechanisms should exist for each trial to register and report SAEs, in particular deaths. Given all most all of these 23 studies included are phase I, which was focusing on the safety, feasibility, and tolerance with few patients, there are insufficient data from phase 2 trials to evaluate the efficacy of stem cells on each respiratory disease.

In conclusion, our study show that independent of the nature of lung diseases and the cell-based therapy, this study suggests that cell therapies may be safe and do not worsen gas exchange, spirometry, the quality of life, cardiopulmonary circulation, and immune system of lung diseases. Deaths of ARDS patients treated with MSC cells are numerically less than controls. Our results support the need for phase 2 and 3 clinical trials to evaluate the long-term impact on these respiratory diseases. Phase 2 and 3 trials will also provide more information on safety of individual cell therapy approaches, doses, and sources in different acute and chronic pulmonary diseases that cannot be adequately assessed in phase 1 trials alone.

## MATERIALS AND METHODS

We conducted our systematic review and meta-analyses in accordance with the methods recommended in the PRISMA guidelines.

### Selection of clinical trials

Three independent investigators searched the potential studies in PubMed, Web of Science, Embase, Cochrane Library, CNKI, and ClinicalTrials.org by July 2016, using the search strategy: (lung OR respiratory OR pulmonary OR airway OR bronchial OR bronchiectasis OR bronchitis OR pneumonia OR silicosis OR asthma OR ALI OR ARDS OR COPD OR cystic fibrosis OR bronchiolitis OR bronchopulmonary dysplasia OR emphysema OR pneumocystosis) AND (safety [TI] OR trial [TI]) AND (stem [TI] OR cell therapy [TI] OR cell-based [TI] OR cellular [TI] OR mesenchymal [TI] OR stromal [TI] OR progenitor [TI]). When the conflicts over inclusions appeared, Dr. Ji was consulted. Our search was not limited by language, race, age, sex, route, study design, phase, and preparation of cells. Two functions of PubMed, “Similar articles” and “Cited by PubMed Central articles” were applied to find additional studies.

### Data extraction

Both individual patient-level data and summarized estimates were extracted by ZLS and HLJ, as we described previously [30]. The marked data were used in meta-analysis. For case series [12, 15–17, 20, 21, 23, 27, 31], we combined individual data using the formulas, $\bar{X}=\frac{\sum x}{n}$ and $\text{SD}=\sqrt{\frac{\sum {\left(x-\bar{x}\right)}^{2}}{n-1}}$, where x stands for variance, $\bar{X}$ is the mean of pooled individual data, and n is the sample size. To combine data of subgroups, mean and SD were computed with these equations: $\text{mean}=\frac{{\text{N}}_{1}{\text{M}}_{1}+{\text{N}}_{2}{\text{M}}_{2}}{{\text{N}}_{1}+{\text{N}}_{2}}$ and $SD=\sqrt{\frac{\left({\text{N}}_{1}-1\right){\text{SD}}_{1}^{2}+\left({\text{N}}_{2}-1\right){\text{SD}}_{2}^{2}+\frac{{\text{N}}_{1}{\text{N}}_{2}}{{\text{N}}_{1}+{\text{N}}_{2}}\left({\text{M}}_{1}^{2}+{\text{M}}_{2}^{2}-2{\text{M}}_{1}{\text{M}}_{2}\right)}{{\text{N}}_{1}+{\text{N}}_{2}-1}}$, where N and M represent the size and mean of subgroup, respectively. If the data was represented as mean and standard error (SE) [12, 21], SE values were converted to SD by this function, $SD=SE\times \sqrt{N}$. For the studies reporting baseline values and net changes in variables [9, 13, 21], we calculated absolute changes using RevMan v5.3. For variables without SD [19, 25], SD value of similar studies for the same parameter was borrowed. If we only know the percent change from baseline [9], then we got the baseline values from other study about the same disease as reference. When we analysis in subgroup of disease, the baseline value and the minimum or maximum value after cell therapy was collected, and only the value of cell therapy group (CTG) was used in studies with controls. On the other hand, when we analysis in subgroup of study design, then the value of change from baseline was used in studies with controls.

All eligible studies met the following criteria. Patients were clinically diagnosed with pulmonary diseases, including ALI/ARDS, COPD, IPF, BPD, silicosis, sarcoidosis, pulmonary tuberculosis, and PAH. The transplanted cells were cells with no restrictions in terms of origin, dose, preconditioning/programming, and route. Publications were original studies. Outcomes were safety and tolerance that were expressed or could be converted or digitized to mean ± SD. The following studies were excluded: conference abstracts or unavailable full articles, absence of detail results and methods, and those reporting therapy for cell transplantation-induced disorders.

### Bias of the included studies

Publication bias between selected studies was detected with the visual symmetry of funnel plots [32] (Supplement 3). Other biases were assessed by bias evaluation table provided by the Cochrane Handbook for Systematic Reviews of Interventions (Supplements 4-5). All of the bias in four studies [9, 10, 13, 14] were low risk. The bias of random sequence generation and allocation concealment were high risk in four studies [26, 28, 29, 31]and unknown in other studies while other bias were low risk in all studies. Other biases were assessed using RevMan according to Cochrane Handbook for Systematic Reviews of Interventions. Of note, there are no regulatory mechanisms for each individual trial to control and report true data of SAEs.

### Meta-regression analysis of SAEs and dose

The total cells received per patients were computed as the product of cells per delivery and the total times. If cells were given per body weight, the total amount of cells was the product of single dose, body weight (60kg), and how many times delivered. Both the association of total cells delivered and total SAEs (Supplement 6) as well as deaths was computed with Stata and software R.

### Statistical analysis

All 23 studies were included for systematic reviews of characteristics of populations. The adverse events (AEs) were grouped by total SAEs, death, and non-fatal SAEs according to the Common Terminology Criteria for Adverse Events v4.0 (CTCAE). Multiple SAEs of one patient counted once. We compared the frequency of incidence rate, Peto OR, RD, and 95% confidence intervals (95% CI) using either fixed-effects or random-effects model. The Mantel-Haenszel OR and RR were used for the sensitivity analyses. The potential side-effects of cell therapy on laboratory and clinical variables were computed as MD and 95% CI using RevMan v5.3. Heterogeneity of extracted data was assessed with the Cochran's Q statistic as the p value and I-square statistic (I^2^) in the pooled analyses, representing the percentage of total variation between studies [32]. If the p value was less than 0.05, or the I^2^ value was greater than 50%, overall estimates were analyzed with the random-effects model. Otherwise, the fixed-effects model was applied. If the p value is significantly different in random-effects model and fixed-effects model, we selected random-effects model to avoid false positives even though the I^2^ value was less than but near 50%. To compute differences in AEs and alterations in laboratory and clinical assays between controlled and uncontrolled studies, we performed χ2 tests and two-sample t-test. Furthermore, we compared the potential differences in these variables between six controlled and 17 noncontrolled studies. To compare differences in SAEs caused by route, source, preconditioning/reprogramming, and dose, we computed OR, Peto OR, RR, and RD.

## SUPPLEMENTARY MATERIALS FIGURES AND TABLES



## Abbreviations

ARDS: acute respiratory distress syndrome
BPD: bronchopulmonary dysplasia
PAH: pulmonary arterial hypertension
XDRTB: extensively drug-resistant tuberculosis
COPD: chronic obstructive pulmonary diseases
IPF: idiopathic pulmonary fibrosis
MSCs: mesenchymal stem (stromal) cells
SAEs: serious adverse events
RCT: randomized controlled trials
nRCT: nonrandomized trials with controls
BM-MSCs: bone marrow derived MSCs
AD-MSCs: adipose-derived MSCs
UBC-MSCs: human umbilical cord-derived MSCs
EPCs: endothelial progenitor cells
G-CSF: granulocyte-colony stimulating factor
OR: odds ratio
RD: risk difference
MD: mean differences
CTG: cell therapy group
FEV1: forced expiratory volume in the first second
FVC: forced vital capacity
IL-6: interleukin 6
IL-8: interleukin 8
SPD: surfactant protein D
CRP: C-reactive protein
LHS: length of hospital stay
VFD: ventilator-free days
SOFA: Sequential Organ Failure Assessment
LIS: lung injury score
6MWD: six-minute walk distance
DOI: duration of intubation
DPAP: duration of nasal continuous positive airway pressure
RSS: respiratory severity score
RR: risk ratio
SD: standard deviation
SE: standard error
