# Supplementary material for: Serious adverse events of cell therapy for respiratory diseases: a systematic review and meta-analysis
Source: Oncotarget. 2017 Feb 16;8(18):30511–23. doi: 10.18632/oncotarget.15426 (PMC5444761; doi:10.18632/oncotarget.15426)
Supplement: Supplementary file 1 [file oncotarget-08-30511-s001.pdf]

Serious adverse events of cell therapy for respiratory diseases: a systematic review and meta-analysis

Supplementary Material

Supplement 1: Comparison of ARDS-associated SAEs between controlled and uncontrolled studies.

	Controlled (events/total)	Uncontrolled (events/total)	OR (95%CI)	RR (95%CI)	RD (95%CI)	Peto OR	p value (OR)
**Total SAEs**	243 (7/25)	333 (4/12)	0.78 (0.18, 3.43)	0.84 (0.30, 2.32)	-0.05 (-0.37, 0.27)	0.78 (0.18, 3.45)	0.78
**Death**	40 (1/25)	250 (3/12)	0.13 (0.01, 1.36)	0.16 (0.02, 1.38)	-0.21 (-0.47, 0.05)	0.12 (0.01, 1.07)	0.09
**Non-fatal SAEs**	240 (6/25)	83 (1/12)	3.47 (0.37, 32.74)	2.88 (0.39, 21.32)	0.16 (-0.07, 0.39)	2.70 (0.48, 15.29)	0.28

Supplement 2: Comparison of PAH-associated SAEs between controlled and uncontrolled studies.

	Controlled (events/total)	Uncontrolled (events/total)	OR (95%CI)	RR (95%CI)	RD (95%CI)	Peto OR	p value (OR)
**Total SAEs**	0 (0/15)	200 (4/20)	0.12 (0.01, 2.38)	0.15 (0.01, 2.52)	-0.20 (-0.40, -0.00)	0.15 (0.02, 1.17)	0.16
**Death**	0 (0/15)	150 (3/20)	0.16 (0.01, 3.38)	0.19 (0.01, 3.38)	-0.15 (-0.33, 0.03)	0.16 (0.01, 1.64)	0.24
**Non-fatal SAEs**	0 (0/15)	50 (1/20)	0.42 (0.02, 11.03)	0.44 (0.02, 10.05)	-0.05 (-0.19, 0.09)	0.17 (0.00, 9.12)	0.60

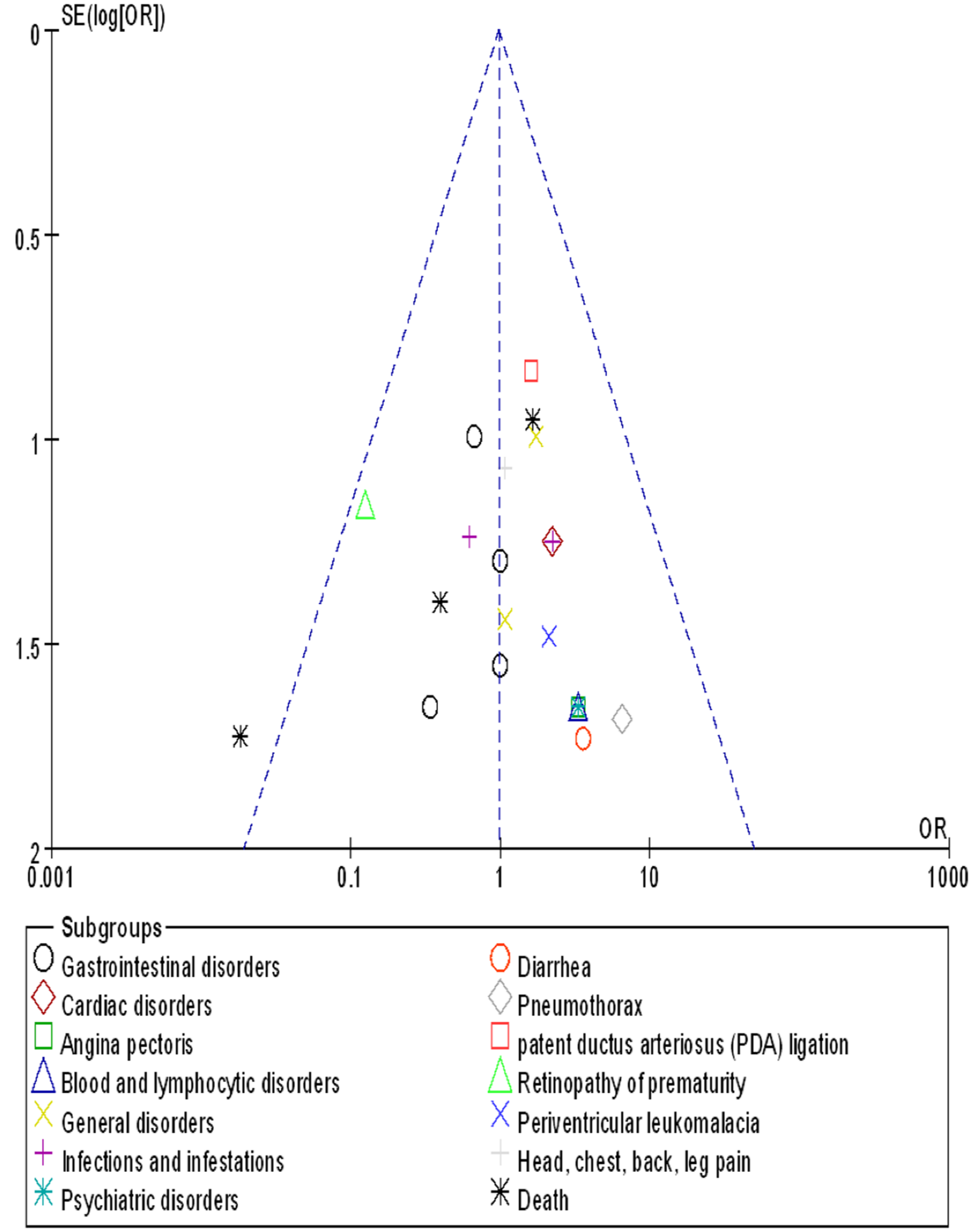

Supplement 3: Analysis of publication bias with all adverse events.

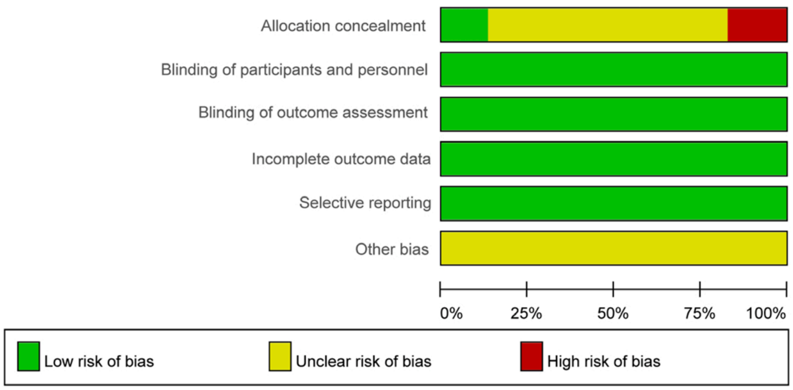

Supplement 4: Risk of bias analysis illustrating the proportion of studies with each of the judgements ('Yes', 'No', 'Unclear') for each entry in the tool. Random sequence generation and allocation concealment are not reported very well and most of them are unclear (<25% are low risk of bias). In contrast, blinding of participants and personnel and outcome assessment, incomplete outcome data, and selective reporting indicate low risk of bias.

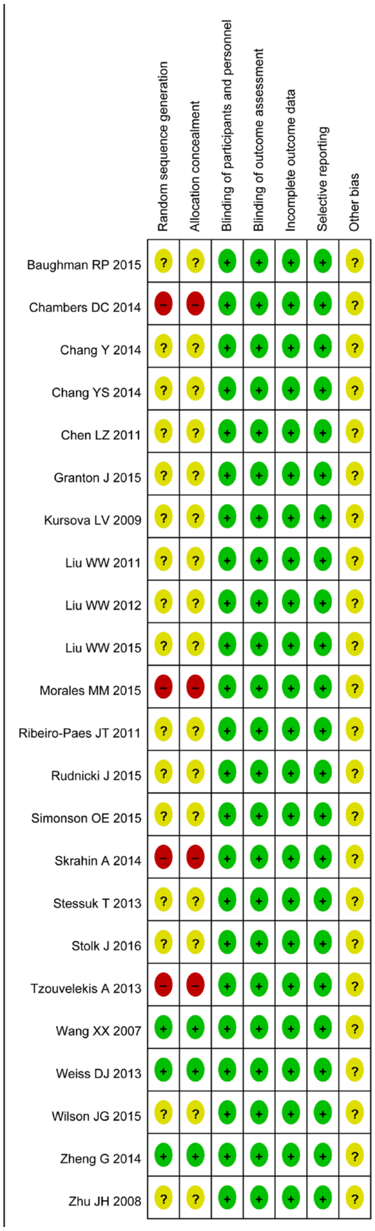

Supplement 5: Risk bias summary of all clinical studies, presenting all of the judgements in a cross-tabulation of study by entry.

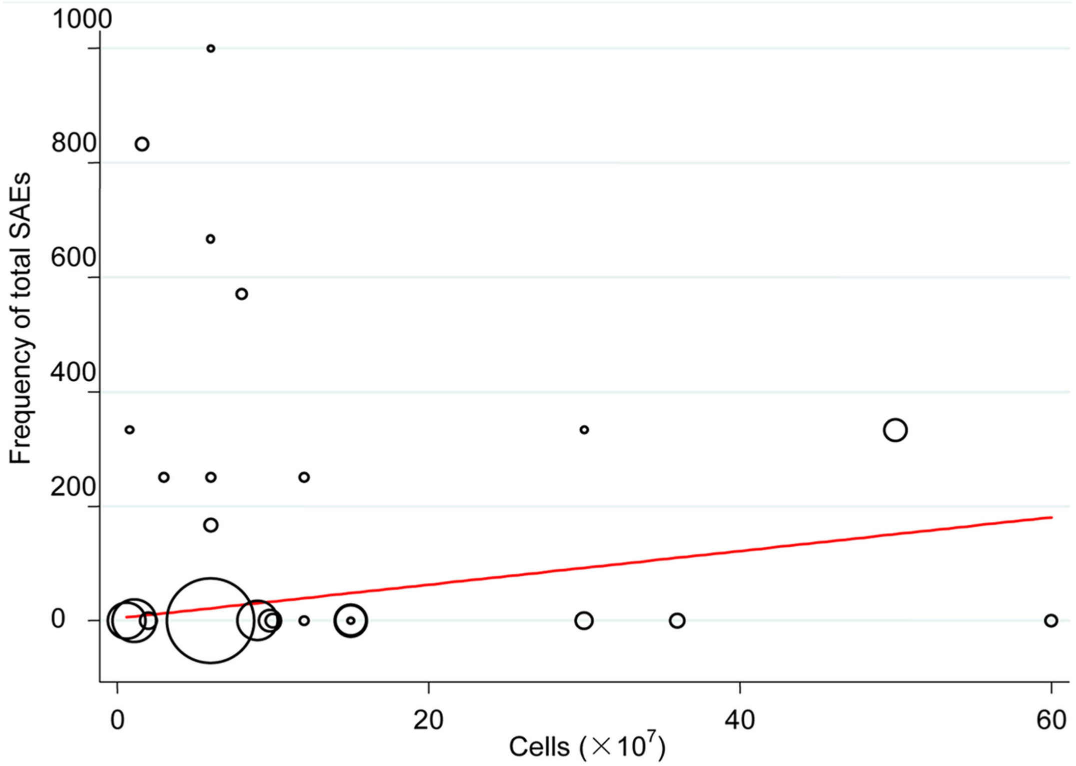

Supplement 6: Analysis of total SAEs as a function of weighed dose (total cells delivered per patient).
